# A Reusable PZT Transducer for Monitoring Initial Hydration and Structural Health of Concrete

**DOI:** 10.3390/s100505193

**Published:** 2010-05-25

**Authors:** Yaowen Yang, Bahador Sabet Divsholi, Chee Kiong Soh

**Affiliations:** School of Civil and Environmental Engineering, Nanyang Technological University, 50 Nanyang Avenue, 639798, Singapore; E-Mails: sabe0002@ntu.edu.sg (B.S.D.); csohck@ntu.edu.sg (C.K.S.)

**Keywords:** electromechanical impedance (EMI), structural health monitoring (SHM), PZT, hydration, concrete, reusable

## Abstract

During the construction of a concrete structure, strength monitoring is important to ensure the safety of both personnel and the structure. Furthermore, to increase the efficiency of *in situ* casting or precast of concrete, determining the optimal time of demolding is important for concrete suppliers. Surface bonded lead zirconate titanate (PZT) transducers have been used for damage detection and parameter identification for various engineering structures over the last two decades. In this work, a reusable PZT transducer setup for monitoring initial hydration of concrete and structural health is developed, where a piece of PZT is bonded to an enclosure with two bolts tightened inside the holes drilled in the enclosure. An impedance analyzer is used to acquire the admittance signatures of the PZT. Root mean square deviation (RMSD) is employed to associate the change in concrete strength with changes in the PZT admittance signatures. The results show that the reusable setup is able to effectively monitor the initial hydration of concrete and the structural health. It can also be detached from the concrete for future re-use.

## 1. Introduction

The hydration of cement is a complicated physical and chemical process which determines the microstructure of the concrete. Various methods have been developed to monitor and characterize the hydration of cementitious materials [1]. During the construction of a concrete structure, strength monitoring is important to ensure the safety of both personnel and the structure itself. The collapse of several structures during their construction highlights the importance of early concrete strength monitoring [2]. Furthermore, to increase the efficiency of *in situ* casting or precasting of concrete, determining the optimal time of demolding is very important for concrete suppliers. In the first few hours after mixing, the fresh concrete gradually achieves solid properties with reasonable compressive strength. Due to different type and amount of cementitious materials, concrete additives (e.g., retarder, deicing) and curing temperature, different rates of hardening are expected. In addition, some other factors like the quality of the cementitious materials further increase the uncertainty in determining the appropriate time for demolding of concrete.

The ultrasonic technique, which needs to have access to both sides of the structure, has been employed to monitor the hydration process [3–6], and any gap because of shrinkage or framework movement can significantly affect the results. A surface bonded PZT transducer has been used for hydration monitoring by Shin *et al*. [7]. However their method could not monitor the early hydration. The concrete needed to be hardened, followed by surface drying and one more day for hardening of the epoxy. This means that the first three days of hydration cannot be monitored. Concrete is the most widely used construction material because it is relatively strong and cheap to produce. The notion of embedded PZT transducers for ultrasonic application or as smart aggregates have been reported in the literature [1,2]. For these methods, at least two PZT transducers, one as sender/actuator and the other as receiver/sensor need to be permanently buried inside the concrete. PZT transducers are cheap but compared to concrete materials, they are still expensive. For repetitive applications, such as in the precast industry, a method to reuse the PZT transducers is essential. Furthermore the buried transducers are not sensitive enough for localized monitoring and the wire connections can easily be damaged during casting, due to framework movement or shrinkage cracks. Other methods such as thermal analysis, x-ray diffraction and scanning electron microscopy, non-contact electrical resistivity and dielectric constant measurement have been reported in the literature [8,9].

Based on the electromechanical impedance (EMI) technique, surface bonded PZT transducers have been widely used for damage detection and parameter identification for various engineering structures over the last two decades [10–19]. In the EMI method, the electromechanical admittance signatures of the PZT acquired at different time are used to calculate the damage indicator [15]. The one dimensional EMI model was developed by Liang *et al*. [20] as follows:

(1) $$\bar{Y}=(\omega j)\frac{\mathrm{wl}}{h}\left[\left({\bar{\varepsilon }}_{33}^{T}-d_{31}^{2}\bar{E}\right)+\left(\frac{Z_{a}}{Z+Z_{a}}\right)d_{31}^{2}\bar{E}\left(\frac{\text{tan}\;\kappa l}{\kappa l}\right)\right]$$

where Z_a_ is the short circuit mechanical impedance of PZT and Z is the mechanical impedance of structure. *Ē* = *E*(1 + η*j*) is the complex modulus of elasticity and ${\bar{\varepsilon }}_{33}^{T}={\bar{\varepsilon }}_{33}^{T}(1-\delta j)$ is the complex electric permittivity at constant stress and $j=\sqrt{-1}$, η and δ are the mechanical loss factor and dielectric loss factor, respectively. *κ* is the wave number, related to angular frequency of excitation *ω* by $\kappa =\omega \sqrt{\rho /\bar{E}}$, where ρ is density of PZT. The electromechanical admittance signatures *Ȳ* consists of real and imaginary parts, the conductance (real part) and susceptance (imaginary part). Conductance has been traditionally used for structural health monitoring due to its better indication of changes in the structure. The prominent effects of structural damage or materials characteristic change on the PZT admittance signatures are the lateral and vertical shifting of the baseline or appearance of new peaks in the signatures, which are the main indicators for damage or material change.

Statistical techniques such as root mean square deviation (RMSD) [10] have been employed to associate the damage or material changes with the changes in the PZT admittance signatures:

(2) $$\mathrm{RMSD}(\%)=\sqrt{\frac{\sum_{i=1}^{N}{\left(G_{i}^{1}-G_{i}^{0}\right)}^{2}}{\sum_{i=1}^{N}{\left(G_{i}^{0}\right)}^{2}}}\times 100$$

where $G_{i}^{0}$ is the baseline signature of PZT conductance, and $G_{i}^{1}$ is the corresponding conductance for each monitoring time at the *i^th^* measurement point. Generally, larger difference between the baseline signature and the subsequent signatures would result in bigger value of RMSD.

In this work, a reusable PZT transducer setup for monitoring hydration of concrete and structural health is developed, where a piece of PZT is bonded to a piece of aluminum or plastic enclosure with two bolts tightened inside the holes drilled in the enclosure. During concrete casting, the bolts and the bottom surface of the enclosure is set to penetrate part of the fresh concrete. At different stages of the first 48 hours after casting, the PZT admittance signatures are acquired. RMSD indices are calculated to associate the change in concrete strength with changes in the PZT admittance signatures. For the three test samples, the reusable PZT setups are not removed from the structure after curing of concrete. The samples are further tested under gradually increasing compressive loading to examine the capability of the reusable setup for structural health monitoring (SHM). The results show that the developed reusable setup is able to effectively monitor the initial hydration of concrete and its structural health. It can also be detached from the concrete for future re-use.

## 2. Experimental Study

### 2.1. Reusable PZT Setup

The acquired signature of the PZT is sensitive to moisture and will change if the PZT is submerged in water. In addition, the enclosure in which the PZT is bonded on needs to be strong enough such that it can be easily detached from the hardened concrete without any damage to the PZT or the enclosure. For these reasons, commercial aluminum and plastic enclosures with dimensions of 50 × 45 × 30 mm and a thickness of 1.5 mm have been used. Two holes have been drilled at the bottom of the enclosure and two bolts been tightened inside to ensure a solid connection of the reusable setup to the concrete. After concrete hardening, the two bolts will be unscrewed and the PZT and enclosure will be removed for future applications. Cement hydration residues will remain on the surfaces of the aluminum enclosure, so the repeatability will be slightly affected after a few reuses.

In the reusable sensor setup, there are two holes at the bottom of the enclosure where water may permeate into the enclosure along the bolts, resulting in circuit shortcut during PZT admittance measurement. To avoid this problem, a water proofing agent called Plastic DIP [21] has been used for waterproofing the PZT transducers. This procedure results in much better signature repeatability for repetitive use; however the PZT sensitivity reduces due to the damping effect of the waterproofing layer.

**Figure 1.**
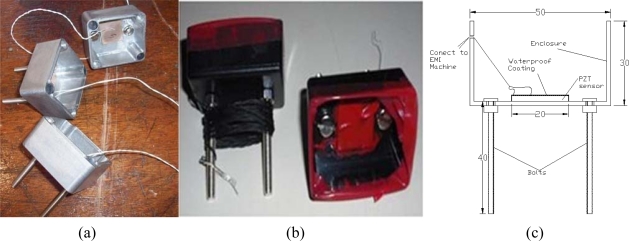
Developed reusable PZT setups.

PZTs with two different sizes of 20 × 20 × 0.5 mm and 20 × 20 × 2 mm developed by PI Ceramic Co. [22] have been used in this experiment. A two-component adhesive, 3M′s DP 460 Epoxy recommended by Smart Material Co. [23] has been used to attach the PZTs to the enclosures. Figure 1(a) shows three developed reusable PZT setups using the aluminum enclosure. Figure 1(b) shows two reusable PZT setups with water proofing coating on the plastic enclosure. And Figure 1(c) illustrates the schematic of the sensor setup.

### 2.2. Initial Hydration Tests

**Figure 2.**
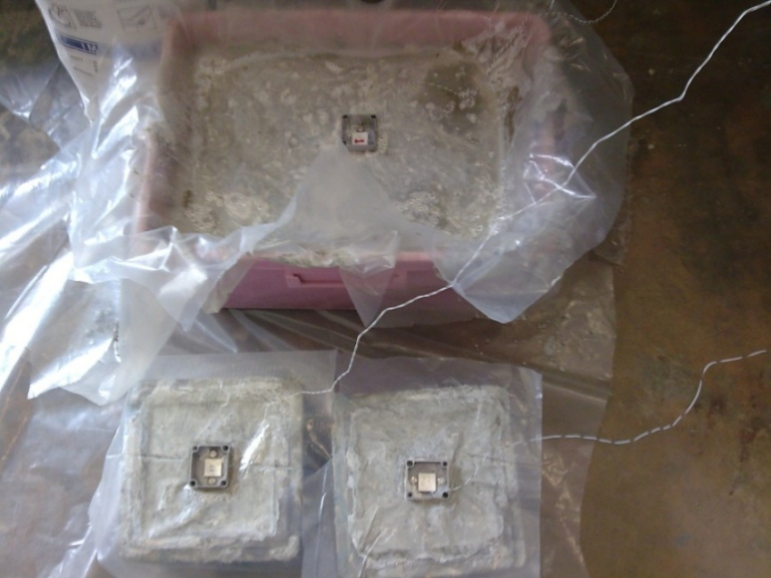
Monitoring hydration rate with reusable PZT transducers in Cast 4.

In this work, the same mix proportion has been cast four times (called Cast 1 to 4) and the same reusable PZT setup called Setup 1a, has been used to evaluate the hydration rate. This process is meant to ensure the reusability of the setup for hydration monitoring. To check the repeatability from one sample to another, in mix Cast 4, a bigger volume has been cast to compare the acquired signatures obtained from different reusable setups. Two more mixes (Casts 5 and 6), one with retarder and the other with faulty retarder which leads to a very weak hydration in the first 48 hours, have also been cast. The results from retarder and faulty retarder specimens are compared to show the ability of the developed setups to distinguish various rates of hydration. The experimental work has been summarized in Table 1. Figure 2 shows the monitoring setup used in Cast 4.

### 2.3. Structural Health Monitoring (SHM)

For the three samples in Casts 7 and 8, the reusable PZT setups were not removed from the structure after curing. These three samples were further tested under normal laboratory compressive loading. Surface bonded PZTs were additionally bonded to the two samples in Cast 8 to compare the sensitivity of surface bonded PZTs with the reusable setups for SHM. The tests were performed in two conditions of cyclic and on-hold loading.

**Figure 3.**
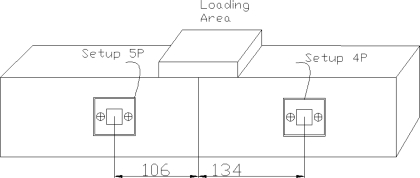
Cycling loading setup for Cast 7.

As shown in Figure 3, for cycling loading, two reusable setups were connected to one rectangular sample with dimensions of 100 × 100 × 500 mm from Cast 7 and neither of them was under direct loading. After recording the initial signatures, the sample was loaded till 10 kN, then the load was released and the PZT admittance signature was acquired. The sample was further loaded to 20 kN, 30 kN and finally till failure, and each time the load was released before recording the EMI signature.

**Figure 4.**
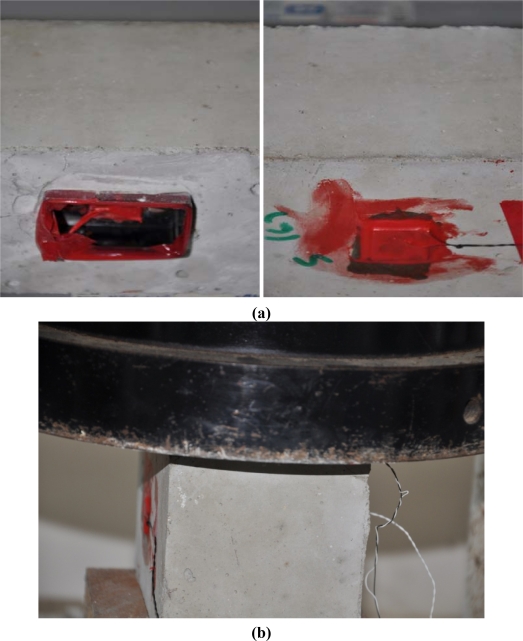
**(a)** Reusable PZT transducer and surface bonded PZT transducer, **(b)** 100 mm cube sample under on hold loading.

**Figure 5.**
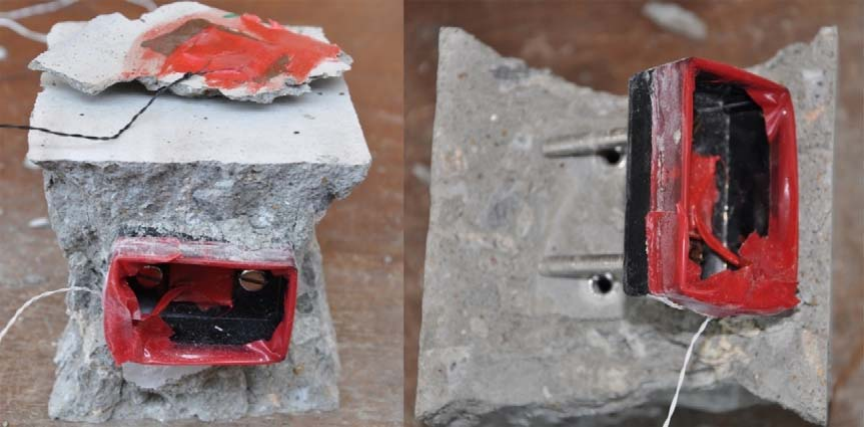
Reusable setup detached after compressive loading.

The two 100 mm cube samples from Cast 8 were tested under on hold loading, *i.e.*, after acquiring the initial signature, the sample was held under 1, 10, 20, 30 kN and till failure and the signatures were obtained without releasing the load. For each sample, one reusable setup and one surface bonded PZT transducer were attached to the structure. Both the reusable setup and the surface bonded PZT were under direct loading area in fairly equivalent distance from the loading point on two opposite sides. Figure 4(a) shows the reusable PZT transducer and the surface bonded PZT on the sample. Figure 4(b) shows one of the samples during on hold compressive loading. Even after failure of the sample, the reusable setup can be detached from the structure without any damage. Figure 5 shows the reusable setup un-screwed and detached from the structure.

## 3. Results and Discussions

### 3.1. Repeatability and Reliability

**Figure 6.**
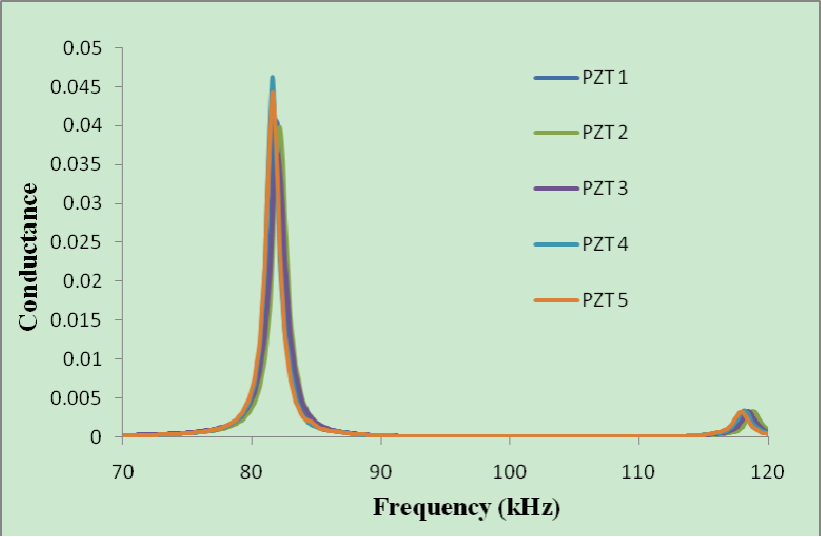
Free signatures obtained from PZT transducers.

Repeatability of the results is a key criterion for reliability of any health monitoring technique. The repeatability of results could be affected by PZT transducers quality, human errors during production of reusable setup including the epoxy thickness and modulus of elasticity and finally damage during the initial hydration or health monitoring process. For all the setups, 2-meter thin wires have been soldered to the PZTs and their signatures have been obtained prior to attaching them to the enclosure. Figure 6 shows the free signatures acquired from five pieces of 2 mm thick PZT transducers. Apparently, the signatures are well repeatable which shows the high quality of the PZT transducers. Although near resonance peak (83 kHz), there is around 10% variation in amplitude.

**Figure 7.**
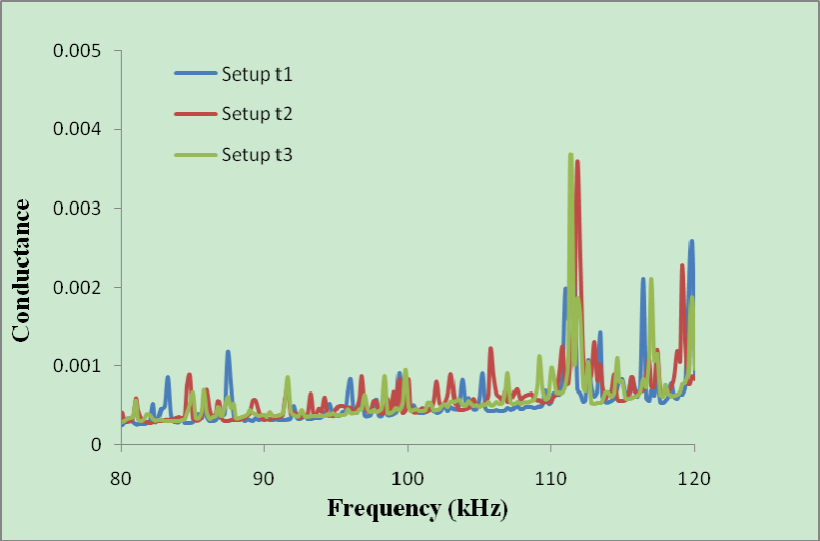
Repeatability of signatures for three setups.

The signatures should also be repeatable in the same initial condition for all the reusable setups. Figure 7 shows the repeatability of the initial signatures for three setups with 0.5 mm thick PZTs. Although the thickness of the epoxy and the quality of bonding may vary from one PZT to another, but with careful preparation, repeatable signatures for different setups can be obtained. For commercial production under controlled environment, repeatable signatures could be obtained. The setups created with 0.5 mm thick PZTs were not used in this work.

**Figure 8.**
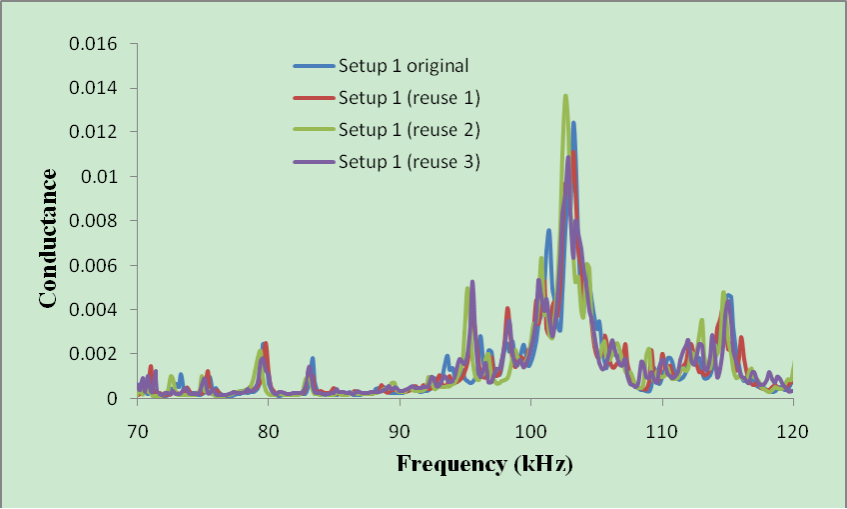
Initial signatures obtained from setup 1a at initial condition and after removing from samples.

The signature obtained at the initial condition should be similar to that obtained after the setup is used and removed from several tested sample. To verify this, Setup 1a has been used four times to check the reusability of the setup. Figure 8 shows the initial signature of this setup and those after the setup was used and detached from several concrete specimens.

As shown in Figure 8, the process of removing the aluminum enclosure from one concrete specimen and reusing it in another specimen has a minor effect on the signatures. The repeatability of signature is very good, even if no water proofing coating was applied on this setup. For the plastic enclosure with waterproofing coating used in Casts 7 and 8, better repeatability has been obtained.

Figure 7 shows the signatures acquired from a thinner PZT (20 × 20 × 0.5 mm) and Figures 8 shows those acquired from a thicker PZT (20 × 20 × 2 mm). It can be seen that the magnitude of the signature for the thicker PZT is around four times of that for the thinner PZT. This indicates that the sensitivity of the thicker PZT is higher than the thinner PZT. As for controlled bonding conditions, the repeatability of the signatures for the thicker PZTs is also better. The above results demonstrate the reliability of developed reusable PZT setup for initial hydration and structural health monitoring of concrete.

**Figure 9.**
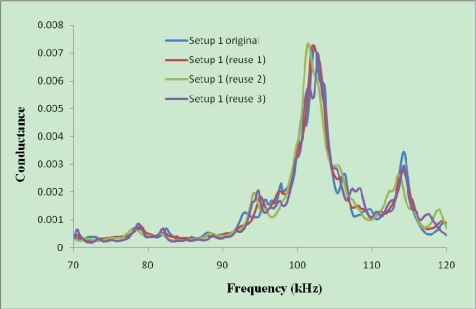
The initial signature obtained from setup after installing inside the concrete.

Furthermore, Figure 9 compares the signatures just after installing the same setup inside different concrete specimens. As can be seen, these signatures are quite repeatable, even though they were obtained from four different mixes cast at different times. Comparing Figures 8 and 9, it is obvious that the magnitude of the signature reduced after the reusable setup was inserted in the fresh concrete, due to the damping effect of the soft fresh concrete.

### 3.2. Monitoring Initial Hydration of Concrete

**Figure 10.**
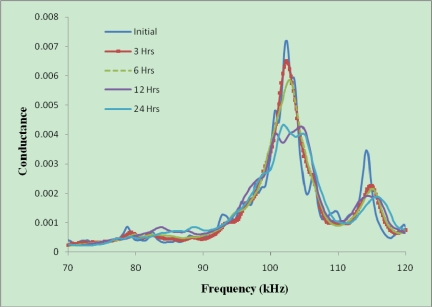
Variation of PZT signatures at different hydration time.

Slump is the measure of the fluidity of concrete. Depending on the amount of water content in the aggregates, the size of aggregates, the temperature during casting and other parameters, the initial slump of concrete can change from one mix to another though the mix proportion is the same. Even the delay time before casting can change the slump of concrete. To eliminate the initial variation of water content and slump from one sample to another, the signatures were obtained from the third hour onwards after casting. After that, the signatures were acquired every three hours. It means that the signatures of the structure at 3, 6, 9,…,48 hours were acquired for comparisons. Figure 10 shows the typical variation of the signatures in the first 24 hours for one concrete specimen, where the initial signature was obtained just after the PZT setup was installed on the mix.

**Figure 11.**
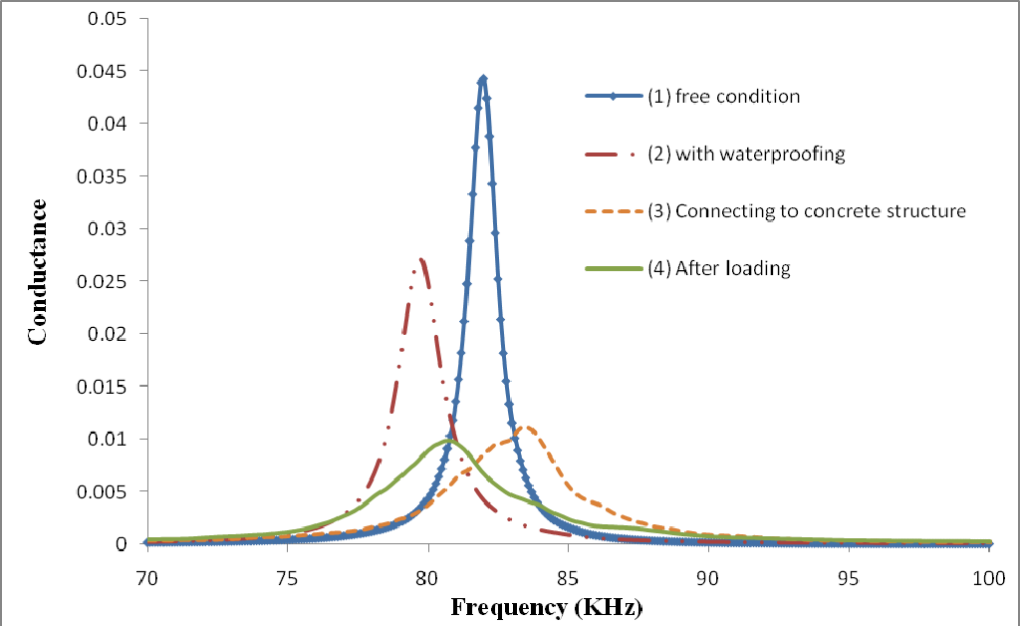
Variation of PZT signatures in different conditions.

As shown in Figure 10, as time passes, the concrete becomes stronger and stronger and, as expected, the signature shifts to the right and the conductance values reduces. Generally, if the host structure becomes stronger, implying less vibration freedom for PZT, the peaks in signature which correspond to natural frequencies of the structure will shift to the right. Also if the PZT is connected to a structure, the magnitude of peaks will decrease as compared to the free PZT signature. On the other hand, if there are cracks or damage in the host structure, the peaks of the signature will shift to the left due to the reduction in the structural stiffness. However, it may not be true for all the peaks in the signature. Figure 11 illustrates the above points. Curve (1) in Figure 11 shows the free signature obtained from 2 mm thick PZT. Curve (2) shows the signature after waterproofing the PZT surfaces (except one surface). Due to the addition of waterproofing, the overall setup became softer and the peak of signature shifted to the left. However, due to the damping effect induced by the water proof coating, the magnitude of peak decreased. Curve (3) shows the signature after installing the PZT setup in the concrete structure. Both stiffness and total mass increased, therefore the peak of signature shifted to right and the magnitude of peak decreased. Finally, Curve (4) shows the EMI signature after loading. There were numerous cracks in the concrete sample which made it weaker, thus the peak shifts to the left.

**Figure 12.**
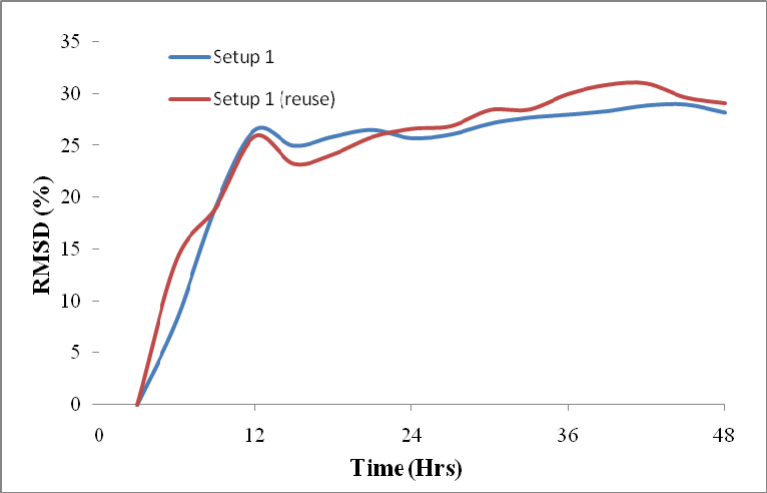
Repeatability of RMSD for same mix proportion.

The RMSD values based on comparison between the signatures measured at different measurement points and the signature at the third hour have been calculated for each mixes. Figure 12 shows the good repeatability of RMSD values for setup 1a in Cast 1 and 2.

As shown in Figure 12, the RMSD value after 12 hours of casting does not change significantly where the baseline signature is measured at the third hour after casting. Thus, for this mix, 12 hours curing was enough to remove the molds.

However, if the RMSD values are calculated based on the difference between two consecutive signatures, the continuous progress of hydration can be observed, as shown in Table 2. The signature at the 24th hour varies by 11.8% as compared to that at the 12th hour, and the signature at the 48th hour varies by 6.12% as compared to that at the 24th hour, though the RMSD compared to the 3rd hour does not change significantly.

**Figure 13.**
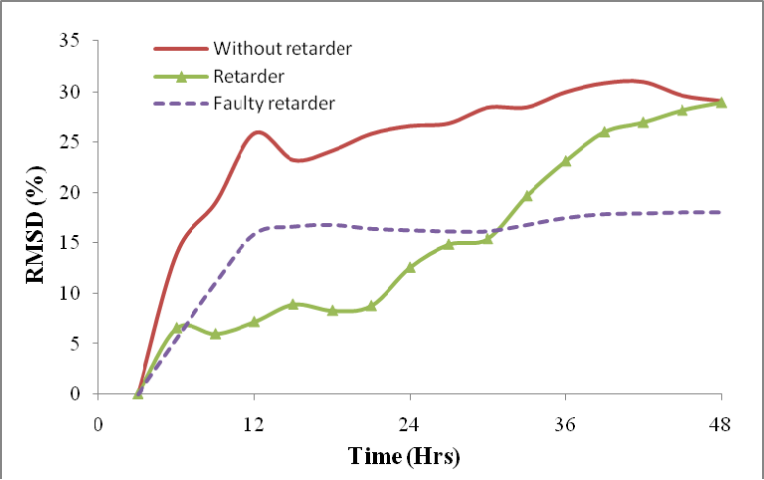
Effect of retarder and faulty retarder on hydration rate.

As mentioned in Section 2.2, two more mixes (Casts 5 and 6), one with retarder and the other with faulty retarder have been cast in order to study the ability of the reusable PZT setup for monitoring different hydration rates. The RMSD changes for these two mixes in comparison to the normal concrete are illustrated in Figure 13.

It can be seen from Figure 13 that the concrete containing retarder gains strength rather gradually, hence to safely remove the molds, the minimum curing time is around 36 hours. For the faulty retarder, the RMSD changes show that the concrete undergoes some initial changes but progresses at much slower hydration rate.

### 3.3. Structural Health Monitoring

As mentioned in Section 2.3, the tests were performed under two conditions of cyclic loading on the sample from Cast 7 and on-hold loading on the two samples from Cast 8. Table 3 shows the summary of the RMSD values for two reusable setups installed in the sample under cyclic loading. Neither of them was under direct loading but setup 5p was slightly nearer to the center of loading.

As shown in Table 3, the RMSD values are approximately in the same range, which shows the repeatability of the reading from one setup to another; and the results are not sensitive to very fine localized cracks very near to the PZT location. This is advantageous for surface bonded PZTs, for which fine cracks very near to the PZT bonding location can cause large RMSD variations which may lead to misinterpretation of the condition of the monitoring region.

For the on-hold loading, both reusable setup and surface bonded PZT transducers were under direct loading area in fairly equivalent distance from the loading point at two opposite sides. Table 4 shows the summary of RMSD values for the two samples.

**Figure 14.**
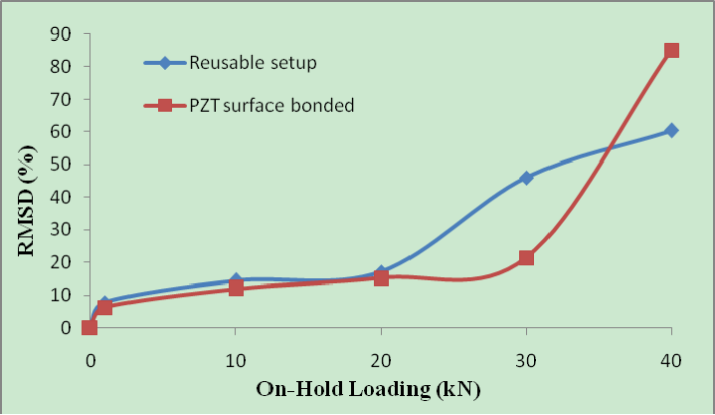
Comparison of RMSD values for reusable PZT setup and surface bonded PZT.

Two signatures were acquired from each setup before loading and RMSD for repetitive reading were around 1% for both the surface bonded PZT and the reusable setup. As shown in Table 4, the reusable setup is very sensitive for SHM and the RMSD values are comparable to those of the surface bonded PZTs. Figure 14 compares the RMSD values for the reusable setup and the surface bonded PZT in sample 1 from Cast 8.

In Figure 14, the RMSD of 30 kN load of the reusable setup is larger than that of the surface bonded PZT, which shows the higher sensitivity of the reusable setup. This is because the reusable setup can monitor both surface and certain depth inside the structure because of the two bolts penetrating into the concrete. For the 40 kN load, the surface bonded PZTs were detached with piece of concrete from the main concrete block (as shown in Figure 5, left) for both samples, causing dramatic change in RMSD values. However, the reusable setup was still in full connection with the host structure. The above results shows that the reusable PZT setup can be used for SHM effectively and it can also be detached from the structure for future application.

## 4. Conclusions

The reusable PZT setup developed in this work is able to effectively monitor the initial hydration of concrete and can be used for SHM. With a controlled bonding layer, the repeatability of PZT signatures has been confirmed and thus the reliability of the reusable PZT setup. It is also found that thicker PZTs have higher sensitivity as compared to the thinner ones. The device can be easily reused for many times with minor changes in sensitivity and initial signature. Various rates of hydration as a result of using retarders were monitored using this reusable setup. The reusable PZT setup showed good consistency in RMSD values as compared to the surface bonded PZT SHM. It can also be detached from the structure for future application.

## Figures and Tables

**Table 1.** Summary of experimental works.

**Casting name**	**Number of samples**	Name & type of sensors * a-Aluminum enclosure * p-Plastic enclosure * Sb- surface bonded PZT	**Purpose**
Cast 1	1	Setup 1a	Study the reusability of the sensor
Cast 2	1	Setup 1a	
Cast 3	1	Setup 1a	
Cast 4	3	Setup 1a, Setup 2a, Setup 3a	Study the repeatability of results from one setup to another
Cast 5	1	Setup 1a	Retarder
Cast 6	1	Setup 1a	Faulty retarder
Cast 7	1	Setup 4p, Setup 5p	Test not directly under loading
Cast 8	2	Setup 4p, Setup 5p, Sb1,Sb2	Test directly under loading

**Table 2.** Continuation of hydration by comparing the signatures in various hours.

**Signature at Time:**	**RMSD change (%) Compared to 3rd Hour:**	**Compared to Hours:**	**Time difference (Hrs)**	**RMSD change (%)**	**Repeatability (%)**

12	26.5	3	9	26.5	0.7
24	25.7	12	12	11.8	0.3
48	28.2	24	24	6.12	0.12

**Table 3.** RMSD values of two reusable PZT setups.

**Loading Level (kN)**	**Setup 4p (RMSD %)**	**Setup 5p (RMSD %)**

0	0	0
10	5.8	9.47
20	7.4	13.62
30	14.65	16.45

**Table 4.** RMSD values for reusable PZT setups and surface bonded PZTs.

**On-Hold Load (kN)**	**Sample 1**		**Sample 2**	

	**Setup 4p**	**Sb1**	**Setup 5p**	**Sb2**

0	0	0	0	0
1	7.73	6.21	7.96	6.95
10	14.34	11.95	10.86	13.19
20	17.04	15.2	39.5	17.29
30	45.96	21.3	59	33.61
40	60.34	85.17	61.51	100.42
